# Supplementary material for: Phenol–Hyaluronic Acid Conjugates: Correlation of Oxidative Crosslinking Pathway and Adhesiveness
Source: Polymers (Basel). 2021 Sep 16;13(18):3130. doi: 10.3390/polym13183130 (PMC8470095; doi:10.3390/polym13183130)
Supplement: Supplementary file 1 [file polymers-13-03130-s001.zip › polymers-1363518-supplementary.pdf]

polymers

MDPI

Supplementary Materials: Phenol-hyaluronic Acid Conjugates: Correlation of Oxidative Crosslinking Pathway and Adhesiveness

Jungwoo Kim Sumin Kim, Donghee Son and Mikyung Shin

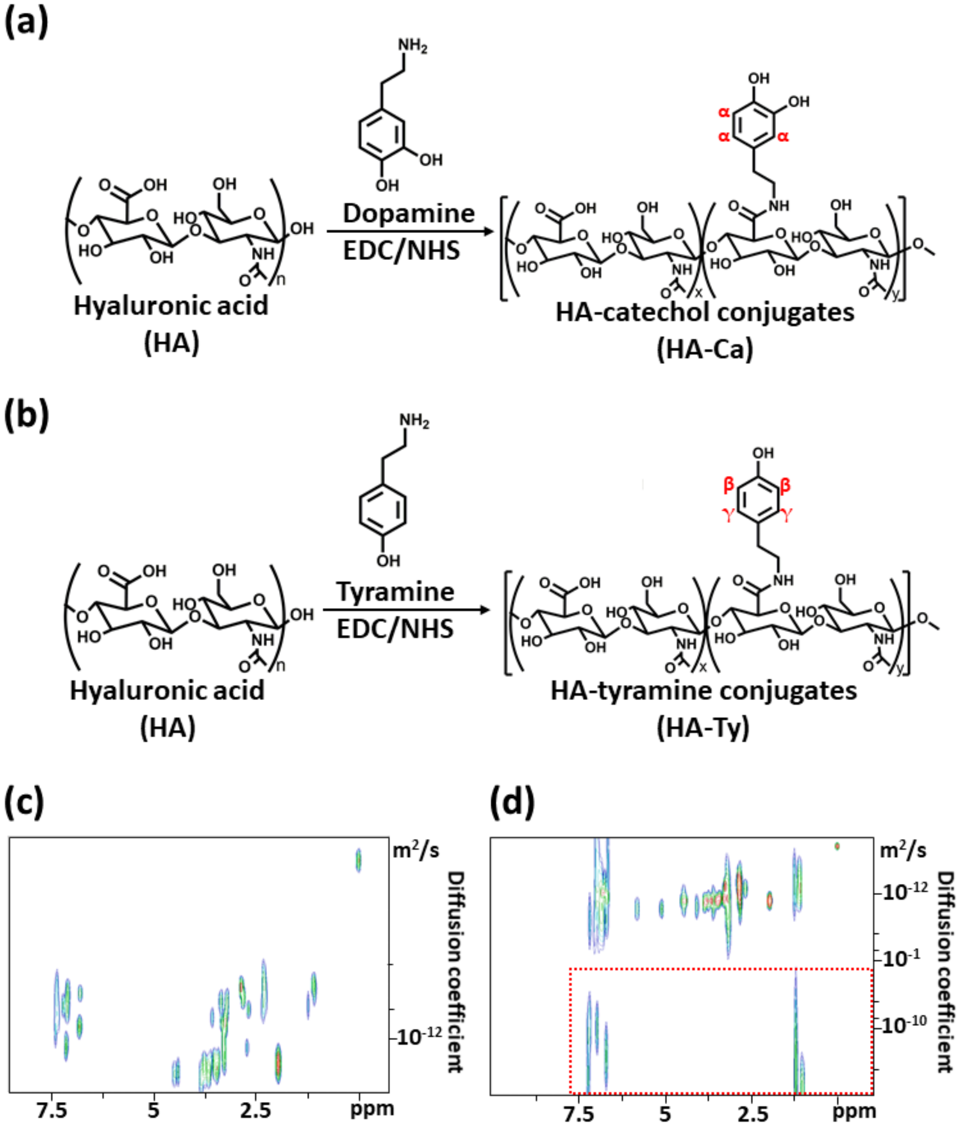

Figure S1. Synthesis and characterization of hyaluronic acid (HA)-phenol conjugates. (**a**) Hyaluronic acid-catechol conjugate (HA-Ca) and (**b**) hyaluronic acid-tyramine conjugate (HA-Ty) synthesized through carbodiimide coupling reaction. DOSY NMR spectra of HA-Ty (**c**) and HA-Ca (**d**) with their diffusion coefficient for evaluation of the sample purity.

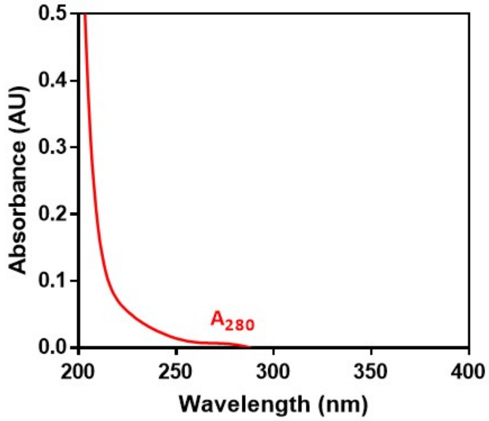

Figure S2. UV-vis spectra of free dopamine (A_{280}) released from the HRP-induced HA-Ca hydrogels.

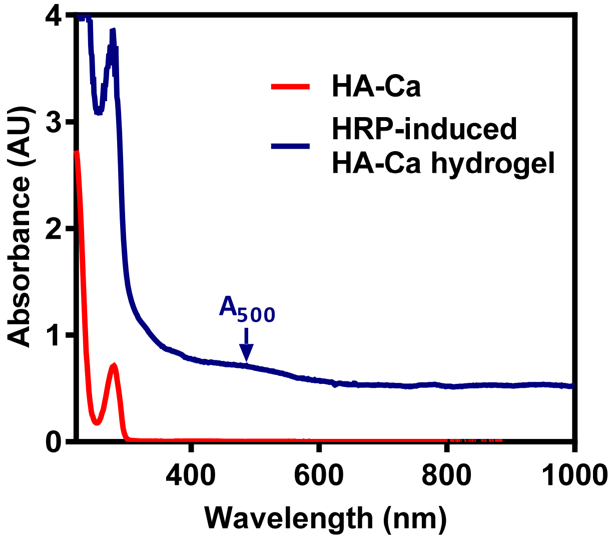

Figure S3. UV-vis spectra of HRP-induced HA-Ca hydrogel.

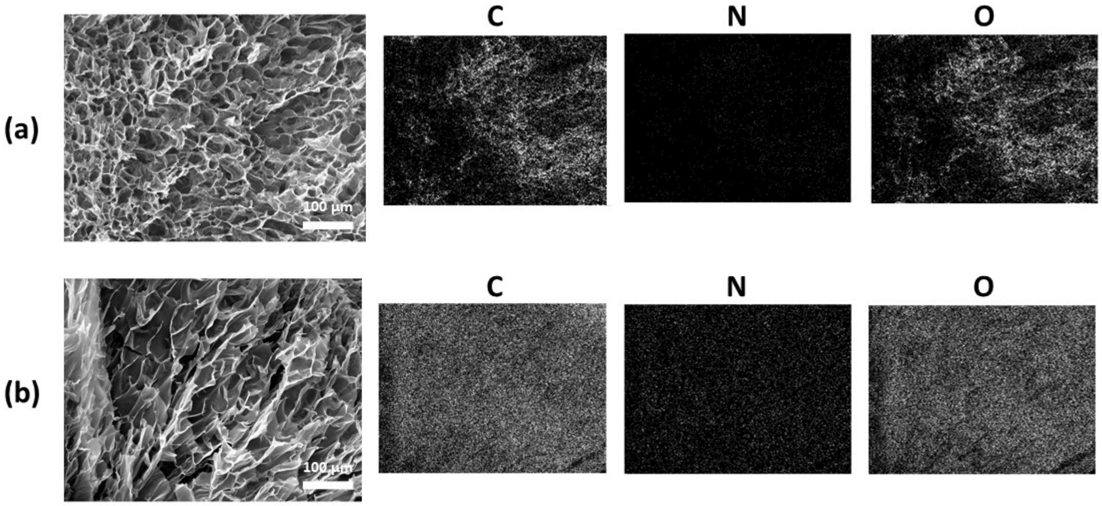

Figure S4. SEM images (1st photos) and EDS mapping (2nd image for carbon (C), 3rd image for nitrogen (N), and 4th image for oxygen (O)) of the lyophilized (**a**) HA-Ca and (**b**) HA-Ty hydrogels crosslinked by HRP/H_2O_2 catalyzed reaction**.**

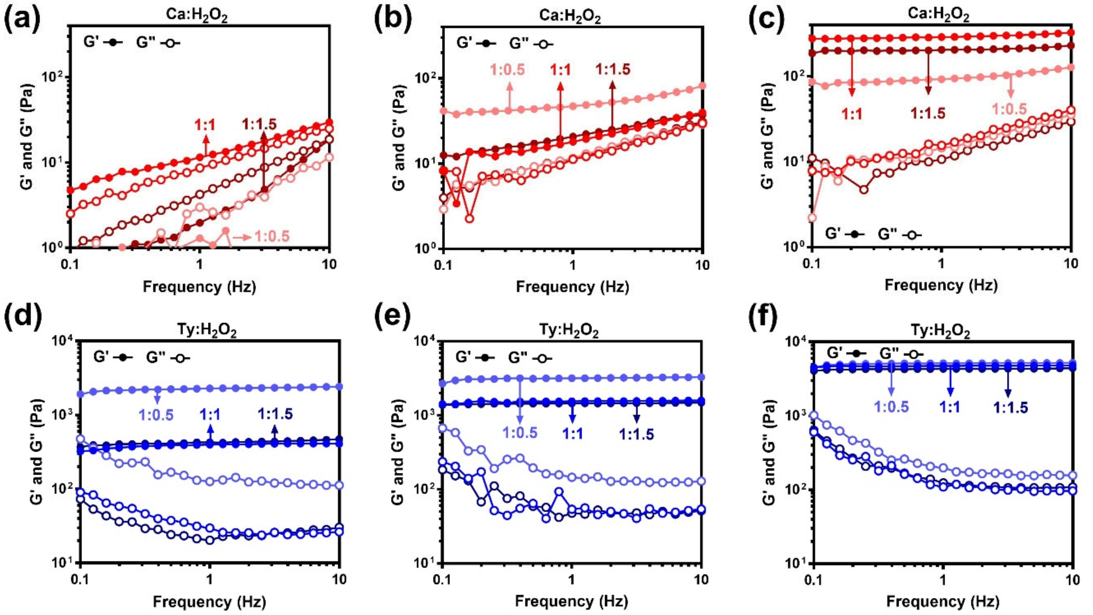

Figure S5. Rheological characterization of HRP-induced HA-Ca hydrogels in different molar ratios of Ca:H_2O_2 at (**a**) 0.1 unit/ml of HRP concentration, (**b**) 0.3 unit/ml of HRP concentration, and (**c**) 0.9 unit/ml of HRP concentration. Rheological characterization of HRP-induced HA-Ty hydrogels in different molar ratios of Ty:H_2O_2 at (**d**) 0.1 unit/ml of HRP concentration, (**e**) 0.3 unit/ml of HRP concentration, and (**f**) 0.9 unit/ml of HRP concentration.

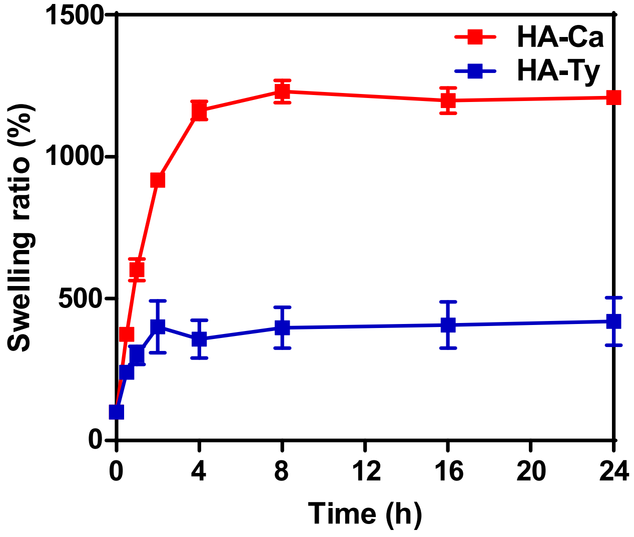

Figure S6. Swelling ratio (%) of HRP/H_2O_2-induced HA-Ca (red) and HA-Ty (blue) hydrogels as a function of time.

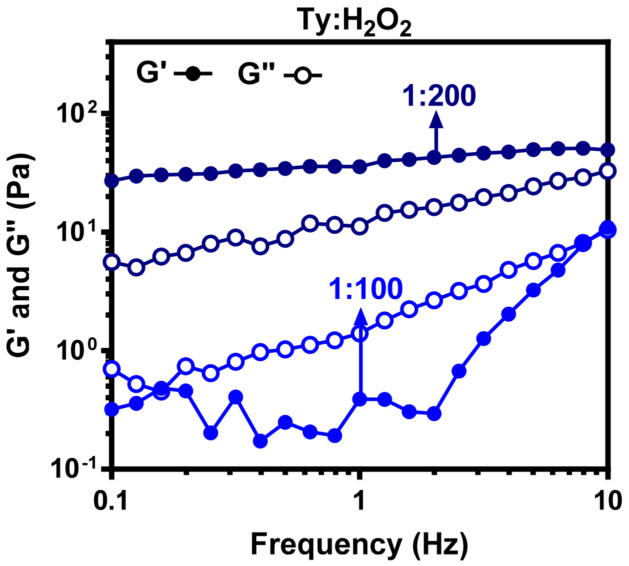

Figure S7. Rheological characterization of APS-induced HA-Ty hydrogels in different molar ratios of Ty:H_2O_2.

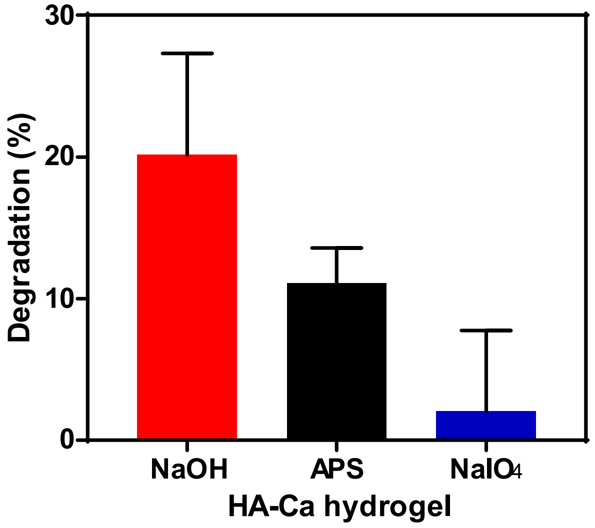

Figure S8. Degradation of HA-Ca hydrogels after swelling of 24 h. The hydrogels prepared in basic condition (pH 10) (red), and under treatment of APS (catechol : APS = 1:100) (black) or $NaIO_4$ (catechol : $NaIO_4$ = 1:1) (blue).
